# New York Letter

**Published:** 1882-06

**Authors:** J. E. Janvrin

**Affiliations:** New York


ARTICLE VI.

NEW YORK, MAY 1, 1882.

DEAR SIR:—Will you kindly call the attention of the readers of your journal to the proposition which I am about to make?

A "System of Gynæcology by American Authors" is in process of preparation, and it is intended that this shall be as nearly encyclopædic as possible.

To me has been allotted the task of writing the chapter on the "History and Statistics of Ovariotomy." To obtain complete statistics of all ovariotomies done in the three-quarters of a century of the history of the operation, is a task quite impossible, as many of the earlier ovariotomists are dead, and their records lost. Even among the living many may be disinclined to co-operate with me as they should. Although the report of each operator's cases may cost him some time and trouble, you will readily see what a valuable fund of information will be obtained by the collation and arrangement of all the facts to be gained.

It is desirable, too, to have all material ready for the press by the end of this year—so that the sooner the returns are made, the lighter will be the task of the editor. Please request all who wish their cases published, to send me the reports before September 1 prox.

May I request your earnest co-operation in the matter, that the work may be worthy the great subject in hand.

The questions to be answered are as follows:

1. Name of Operator? 2. Age of Patient? 3. Nationality? 4. Married or single? 5. Aspiration or previous tapping? 6. Duration of growth? 7. Laparotomy or vaginal Operation?

8. Condition of patient at time of Operation? 9. Were antiseptic precautions used? 10. Was the spray used? 11. Long or short incision? 12. Adhesions or other complications? 13. Double or single Ovariotomy? 14. Pathological features of Cyst? 15. Treatment of the Pedicle? 16. With or without Drainage? 17. Duration of operation? 18. Complicated or uncomplicated history after Operation? 19. Anti-Pyretic used, if any? 20. Result. Cause of death, if any? 21. Primary or secondary operation?

Let the answers be as concise as possible. In many cases a simple yes or no will suffice.

Will you kindly assist in giving the greatest publicity possible to this notice, and publish it for a sufficient length of time in your journal? Blanks, containing lists of the questions referred to, will be sent to any address.

All communications should be addressed to me at 191 Madison Avenue.

J. E. JANVRIN.

---

THE MEDICAL STUDENT'S PRIMER.—What place is this? This is the Pathological Society. How does one know it is the Pathological Society? You know it by the specimens and the smells. What does that gentleman say? He says he has made a post-mortem. All the gentlemen make post-mortems. They would rather make a post-mortem than go to a party. What is that on a plate? That is a tumor. It is a very large tumor. It weighs 112 pounds. The patient weighed eighty-eight pounds. Was the tumor removed from the patient? No, the patient was removed from the tumor. Did they save the patient? No, but they saved the tumor. What is this in the bottle? It is a tapeworm. It is a long tapeworm: it is three-quarters of a mile long. Is that much for a tapeworm? It is, indeed, much for a tapeworm, but not much for the Pathological Society.—MED. REC.

